# Factors Associated with Wearable-Derived Physiological Stress Indicators Among Clinical Nurses: A Longitudinal Observational Study Using Continuous Monitoring

**DOI:** 10.3390/healthcare14152282

**Published:** 2026-07-27

**Authors:** Wan-Yun Hsu, Li-Yun Tsai, Ching-Yi Lai, Jun-Peng Chen, I-Chieh Chen, Da-Ren Chen, Yi-Ming Chen

**Affiliations:** 1 International Medical Service Center, Taichung Veterans General Hospital, Taichung 407219, Taiwan; cutewanyun@gmail.com; 2 Department of Nursing, Central Taiwan University of Science and Technology, Taichung 406053, Taiwan; 108694@ctust.edu.tw (L.-Y.T.); 107943@ctust.edu.tw (C.-Y.L.); 3 College of Nursing, Central Taiwan University of Science and Technology, Taichung 406053, Taiwan; 4 Department of Medical Research, Taichung Veterans General Hospital, Taichung 407219, Taiwan; pippan7676@vghtc.gov.tw (J.-P.C.); icchen@vghtc.gov.tw (I.-C.C.); 5 Department of Information Management, National Taichung University of Science and Technology, Taichung 404336, Taiwan; 6 Division of Allergy, Immunology and Rheumatology, Taichung Veterans General Hospital, Taichung 407219, Taiwan; 7 Graduate Institute of Clinical Medicine, College of Medicine, National Chung-Hsing University, Taichung 402202, Taiwan; 8 School of Medicine, National Yang-Ming Chiao Tung University, Taipei 112304, Taiwan; 9 Department of Post-Baccalaureate Medicine, College of Medicine, National Chung-Hsing University, Taichung 402202, Taiwan; 10 Precision Medicine Research Center, College of Medicine, National Chung-Hsing University, Taichung 402202, Taiwan

**Keywords:** nurses, occupational stress, shift work, wearable electronic devices, physiological monitoring, heart rate variability, occupational health

## Abstract

**Highlights:**

**What are the main findings?**

- Younger age, day-shift work, and fixed (non-rotating) schedules were independently associated with higher scores on the wearable-derived physiological stress indicator, whereas night-shift work and more frequent rotation were associated with lower scores, a counterintuitive pattern that warrants further confirmation.
- A higher daily step count was independently associated with higher physiological stress, suggesting that step count may serve as an objective indicator of clinical workload.

**What are the implications of the main findings?**

- Wearable-based physiological monitoring offers a feasible, objective complement to traditional self-report assessment of nurse stress.
- Occupational-health interventions may benefit from targeting younger, less experienced nurses and those on fixed day schedules who carry the greatest physiological burden.

**Abstract:**

**Background:** Work-related stress affects both nursing care quality and workforce wellbeing, yet conventional self-report assessment is prone to recall bias and is unable to capture real-time fluctuations. This study used a consumer-grade wearable device to continuously monitor physiological stress and identify associated factors among clinical nurses. **Methods:** In this longitudinal observational study, 52 clinical nurses at a tertiary medical center located in Taiwan wore a Garmin Vivosmart 4 over a period of 90 consecutive days. The wearable-derived stress score (0–100), computed from heart rate variability, was then averaged to a daily mean per nurse. A multivariable generalized estimating equation (GEE) model identified independent predictors of daily mean stress. **Results:** Older age was associated with lower physiological stress (β = −0.55 per year of age; *p* = 0.001). Relative to day shift, night-shift work was associated with substantially lower stress (β = −11.97; *p* < 0.001), whereas evening shift work did not differ significantly from day shift work. Two or more shift changes monthly were associated with lower stress than a consistent schedule (β = −14.74; *p* = 0.002). A higher daily step count was associated with higher stress (β = 1.06 per 1000 steps/day; *p* < 0.001). Education, position, and department showed no significant association. **Conclusions:** Younger age, day shift work, a consistent schedule, and a higher daily step count were associated with higher scores on the wearable-derived physiological stress indicator. Wearable-based monitoring may offer a feasible, objective complement to traditional self-report assessment. Our findings require confirmation against validated self-report measures before informing occupational-health decisions.

## 1. Introduction

Work-related stress among nurses has become a critical concern for healthcare systems worldwide, with consequences for both the quality of patient care and the wellbeing of the nursing workforce [1,2]. Nursing inherently entails high responsibility, sustained emotional labor, and complex clinical decision-making, which together render nurses particularly vulnerable to occupational stress [3]. Reported prevalence varies across settings and regions, but a substantial proportion of clinical nurses experience clinically meaningful work-related stress [4,5].

Elevated stress among nurses has far-reaching implications. It has been linked to reduced job satisfaction, higher rates of burnout, and increased turnover intention [6,7], outcomes that affect not only individual nurses but also organizational performance and, ultimately, patient safety [8]. The economic burden attributable to stress-related absence and nurse turnover is considerable [9]. Reducing and managing nurse stress is therefore both a workforce-sustainability and a patient-safety priority.

Despite extensive research, stress in nursing populations has been assessed predominantly through self-report instruments and retrospective questionnaires [10,11]. Although valuable, such measures are susceptible to recall and reporting bias and cannot capture real-time variation in stress across a working shift [12]. Important gaps therefore remain in our understanding of the temporal dynamics of stress and of how specific work-related factors relate to objectively quantified physiological load in authentic clinical settings [13].

Wearable technology offers a novel opportunity to measure and monitor stress objectively through physiological signals [14]. Contemporary devices can track parameters such as heart rate and heart rate variability continuously, providing fine-grained insight into stress patterns during real clinical work [15]. Validation studies have reported reasonable agreement between wearable-derived indices and established psychophysiological markers of stress, supporting their application in occupational-health research [16,17].

Nevertheless, few studies have used wearable devices to examine the relationship between work-related factors and stress among clinical nurses [18], and the influence of shift type, shift rotation, department, and other occupational characteristics on objectively quantified physiological stress remains poorly characterized [19,20]. Prior wearable-based studies in nurses have largely been cross-sectional or limited to short monitoring windows, leaving the temporal dynamics of stress under real clinical conditions insufficiently characterized. The present study addresses these gaps by enrolling clinical nurses for continuous monitoring over 90 days and applying a generalized estimating equation framework that exploits the repeated daily structure of the data. Addressing these gaps is essential for developing evidence-based interventions and scheduling policies that genuinely support nurse wellbeing.

Importantly, occupational stress among nurses is not determined solely by organizational and scheduling factors; it is also shaped by psychological processes, including emotion regulation, coping strategies, resilience, psychological flexibility, and the emotional labor intrinsic to caring work [21]. For example, psychological inflexibility has been shown to fully mediate the relationship between difficulties in emotion regulation and perceived stress among intensive care unit nurses [21], indicating that perceived stress reflects cognitive and emotional appraisal processes that physiological signals capture only indirectly. These processes also contribute to the high prevalence of burnout and emotional exhaustion documented in the nursing workforce worldwide [22,23]. Because such psychological constructs are typically assessed by self-report rather than a wearable-derived physiological stress indicator, the present study therefore investigated the demographic and occupational factors associated with an objective, wearable-derived physiological stress indicator among clinical nurses, using continuous physiological monitoring with a consumer-grade wearable device over a 90-day period. By relying on objective, repeated measurements rather than retrospective self-report, we aimed to identify the factors most strongly associated with physiological stress and to test prevailing assumptions about occupational stressors in nursing practice.

## 2. Materials and Methods

### 2.1. Study Design and Setting

We conducted a longitudinal observational study at a tertiary medical center located in Taiwan between the period of February to August 2022. Physiological stress was monitored continuously using a wrist-worn wearable device, allowing real-time stress to be captured in an authentic clinical environment without experimental manipulation. Because each nurse contributed repeated daily measurements over the monitoring period, the design supports analysis of within-person variation in addition to between-person differences.

### 2.2. Participants and Sampling

A convenience sample of registered nurses was recruited from the surgical and emergency departments. Eligibility criteria were: (1) full-time employment as a registered nurse; (2) willingness to wear the monitoring device continuously throughout the study period; and (3) at least three months of experience in the current clinical position. Nurses were excluded if they were pregnant, had a diagnosed condition likely to affect physiological measurements, or were concurrently enrolled in another study involving physiological monitoring.

The sample size was determined primarily by feasibility and the practical demands of sustained 90-day continuous monitoring rather than by a formal a priori power calculation. Fifty-two eligible nurses were enrolled and were followed for the full 90-day monitoring period. Because each nurse contributed up to 90 daily observations, the effective information available for within-person, time-varying predictors substantially exceeds that implied by the participant count alone; precision for these predictors is conveyed through the reported confidence intervals rather than a pre-specified power target. Between-person contrasts involving small strata (night-shift work, n = 7; managerial, n = 6; postgraduate-educated, n = 4) are correspondingly imprecise and should be interpreted with caution.

### 2.3. Ethical Considerations

The study protocol was approved by the Institutional Review Board of the participating hospital (approval number: IRB-2022-0145, approved on 24 January 2022). Written informed consent was obtained from all participants. All physiological and demographic data were de-identified prior to analysis, and confidentiality was maintained throughout the study.

### 2.4. Physiological Stress Monitoring

Physiological data were acquired using Garmin Vivosmart 4 devices (Garmin Ltd., Olathe, KS, USA) and the Garmin Connect application. This device was selected for its continuous physiological-monitoring capability, lightweight design, extended battery life, and ease of use [14]. The device records heart rate and heart rate variability (HRV) using a photoplethysmography (PPG) sensor, Garmin Elevate™ (Garmin Ltd., Olathe, KS, USA), and an embedded proprietary algorithm (Firstbeat Technologies Ltd., Jyväskylä, Finland) derives a stress score from HRV at three-minute intervals by inferring the balance between sympathetic and parasympathetic activity. The score is reported on a 0–100 scale, with higher values indicating greater physiological stress. To limit the misclassification of physical exertion as psychological stress, the algorithm suppresses stress estimation during periods of detected physical activity and reports a value only during sufficiently stationary periods; residual effects of posture and circadian phase on the output may nonetheless remain. When benchmarked against an electrocardiographic reference, the Garmin Vivosmart 4 stress score has been shown to rise significantly during acute mental-stress tasks and to correlate with heart rate and HRV in the expected directions in healthy young adults, although it corresponds only marginally with self-reported perceived stress [17]. Accordingly, the score is treated here as a wearable-derived physiological stress indicator rather than a measure of perceived stress.

Wear time was monitored through the device’s built-in tracking function. No minimum daily wear-time threshold was applied, and every day on which the device recorded a daily stress score was retained. Physiological data were synchronized to a secured research database through the manufacturer’s application programming interface.

### 2.5. Data Architecture and Preprocessing

**Figure 1:**
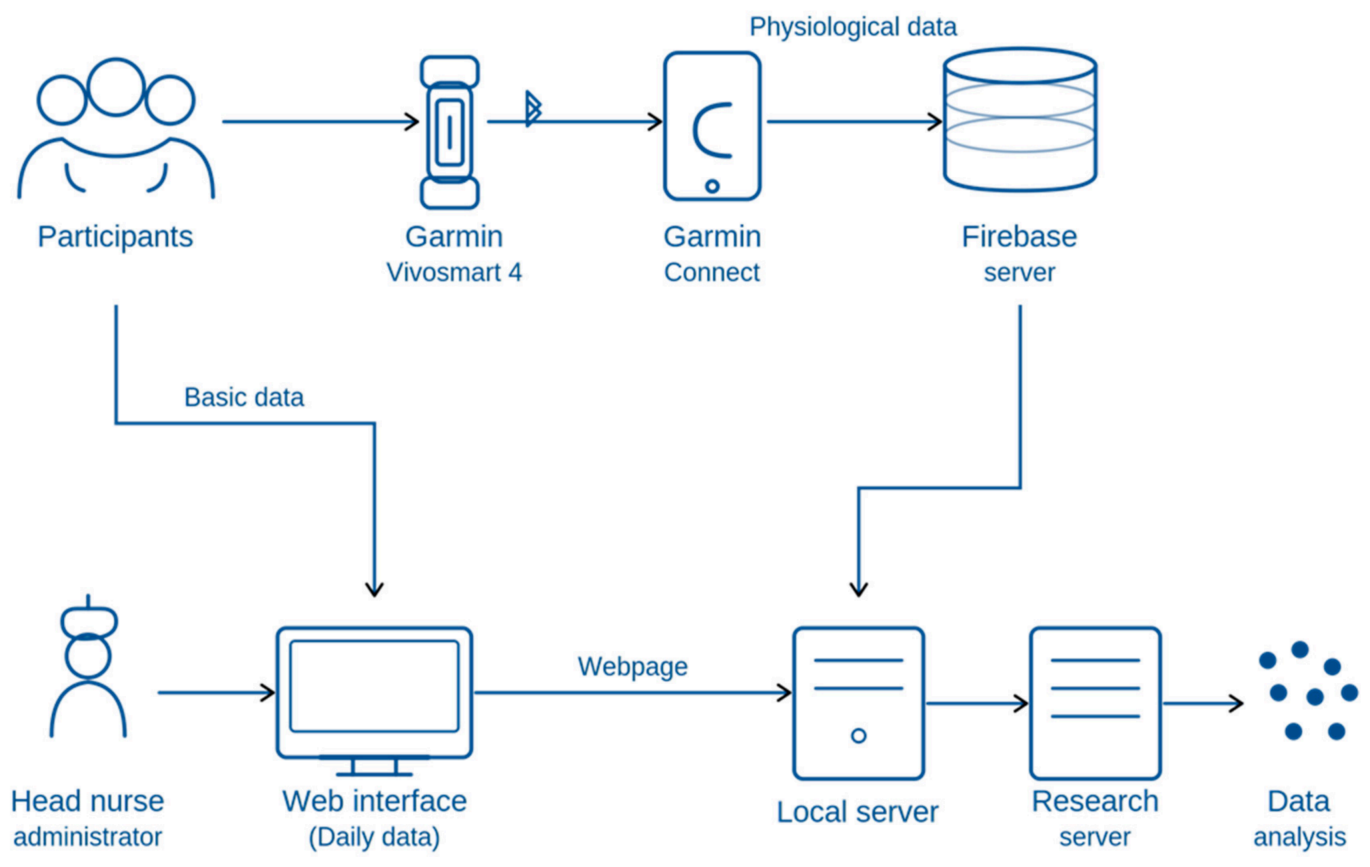
Overview of the data-collection and processing framework.

The data-acquisition system comprises two complementary pathways (Figure 1). In the physiological pathway, each wristband transmitted data via Bluetooth to the participant’s smartphone, which relayed the encrypted data to the manufacturer’s servers; the data were then retrieved from a Firebase data warehouse where it had been placed in storage. In the management pathway, a designated head nurse recorded demographic and work-status information through a web interface connected to a local server. The local server integrated the warehoused physiological data with the occupational records, performed data cleaning and normalization, and ultimately generated outputs accessible through the web interface.

To protect confidentiality, participants were issued randomly generated credentials for device registration so that stored physiological records were linked only to pseudonymous identifiers, with directly identifying information held separately, in compliance with applicable data-protection requirements. Separating the data warehouse from the local server also permitted interim data inspection during the collection period, which was particularly valuable for a longitudinal design.

### 2.6. Demographic and Occupational Data

With participant consent, demographic and occupational information was extracted from institutional records, including age, sex, education level, clinical department, shift type, shift-change frequency, job title, salary, marital status, and parental status (presence of children). Shift-change frequency was categorized as a consistent schedule (no change), one change per month, or two or more changes per month.

### 2.7. Statistical Analysis

Continuous variables were summarized as means with ranges or as medians with interquartile ranges (IQRs), while categorical variables were summarized as frequencies and percentages. For each participant, the wearable-derived stress score was aggregated to a daily mean, yielding repeated daily observations nested within individuals across the 90-day monitoring period. Missing daily observations were handled through available-case analysis, with all available daily stress scores retained in the generalized estimating equation model without imputation.

To account for the correlation among repeated daily measurements within the same nurse, a multivariable generalized estimating equation (GEE) model was fitted, with the daily mean stress score as the outcome and the individual nurse as the subject (clustering) variable. An exchangeable working correlation structure was specified, and robust standard errors were used to ensure valid inference that is relatively insensitive to misspecification of the correlation structure. The exchangeable structure was chosen because repeated daily observations of a nurse have no single dominant temporal-decay ordering and it is parsimonious, requiring a single correlation parameter; the within-subject correlation estimated under this structure was ρ = 0.142. This a priori choice was corroborated empirically: among the candidate working correlation structures, the exchangeable specification yielded the lowest quasi-likelihood information criteria (QIC = 715,376.6; QICC = 715,282.6), below both the independent (QIC = 726,198.3; QICC = 726,096.6) and first-order autoregressive AR(1) (QIC = 716,235.6; QICC = 716,149.9) structures, where smaller values indicate better fit (Supplementary Table S1). Explanatory variables comprised age, shift type, shift-change frequency, job title, education level, and clinical department, together with daily step count and elapsed study time as time-varying covariates. Multicollinearity among these predictors was formally assessed: all covariates had variance inflation factors below 10 (tolerance > 0.1) and the condition index was below 30, indicating no problematic collinearity (Supplementary Table S2). Regression coefficients (βs), standard errors (SEs), 95% confidence intervals (CIs), and *p*-values are reported. A two-sided *p* value below 0.05 was considered statistically significant. Analyses were performed using SPSS version 22.0 (IBM Corp., Armonk, NY, USA).

To assess the robustness of the findings to model specification, we performed sensitivity analyses in which shift type and shift-change frequency were entered separately (Supplementary Table S3).

## 3. Results

### 3.1. Sample Characteristics

A total of 52 registered nurses completed the 90-day monitoring period and were included in the analysis. Their demographic and occupational characteristics are summarized in Table 1. The median age was 28.5 years (IQR 25.0–33.0), and most nurses were aged 21–30 years (61.6%). The cohort was predominantly female (48/52, 92.3%), and the great majority held a bachelor’s degree (92.3%), with only four nurses (7.7%) holding a master’s or doctoral degree. Participants were drawn in approximately equal proportions from the surgical (51.9%) and emergency (48.1%) departments. With respect to work schedule, 53.8% worked day shifts, 32.7% evening shifts, and 13.5% night shifts. Most nurses were single (75.0%) and had no children (82.7%), and the majority held a staff-nurse position (88.5%) rather than a deputy head nurse or head nurse role (11.5%). The median monthly salary was approximately US$1555 (IQR US$1368–US$1839). Across the monitoring period, nurses recorded a median of 7864 steps per day (IQR 5367–11,564). Over the 90-day period, the 52 nurses contributed 3268 valid monitoring days out of a potential 4680 person-days, corresponding to a mean wear-time compliance of 69.8% (SD 22.9%) relative to the 90-day window, with a median of 59.0 valid monitoring days per nurse (IQR 50.3–85.5). No days were excluded on the basis of a fixed daily wear-time threshold. The remaining 30.2% of potential person-days did not yield a usable daily stress score because the device was not worn or no score was recorded. We further examined whether wear-time compliance varied across participant and work characteristics: compliance did not differ significantly by shift type (Kruskal–Wallis test, *p* = 0.773) and was not significantly associated with age (Spearman’s ρ = −0.084, *p* = 0.552) or workload (ρ = 0.133, *p* = 0.347), but it differed by department, being higher among surgical than emergency nurses (median 0.93 vs. 0.60; Kruskal–Wallis test, *p* < 0.001) (Supplementary Table S4).

### 3.2. Distribution of Physiological Stress Scores

**Figure 2:**
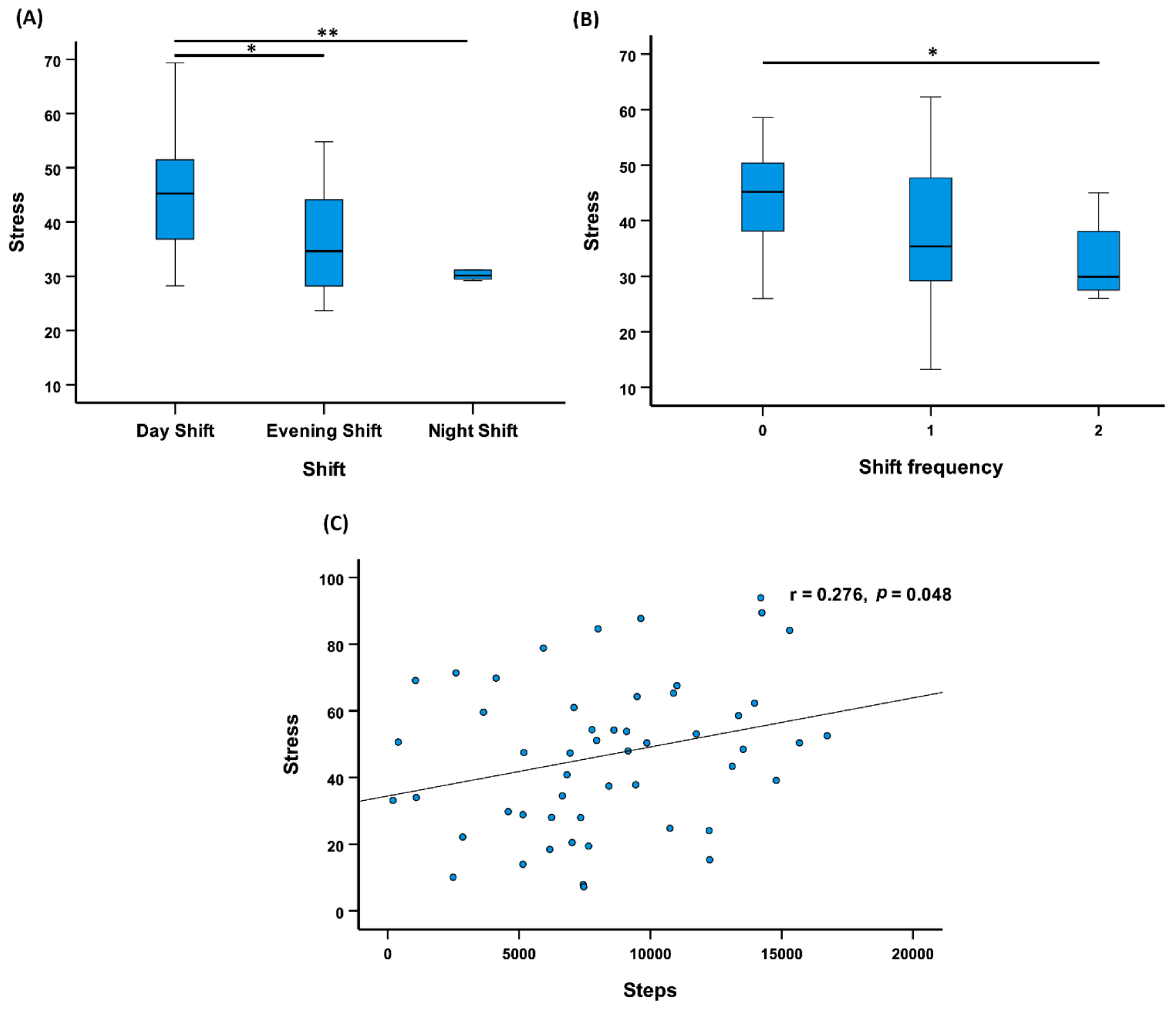
Distribution of daily mean wearable-derived physiological stress scores by (**A**) shift type, (**B**) shift changes per month (0, 1, ≥2), and (**C**) daily step count. (**A**,**B**): Box plots (median, IQR, 1.5× IQR whiskers); * *p* < 0.05, ** *p* < 0.01, Kruskal–Wallis. (**C**): Individual participants, unadjusted trend line.

Figure 2 presents the distribution of the daily mean wearable-derived physiological stress score for three of the four variables that remained independently associated with the outcome in the multivariable model. Each plotted observation represents an individual nurse-day; all panels remain unadjusted. By shift type (panel A), the median score was highest among day-shift nurses, intermediate among evening-shift nurses, and lowest among night-shift nurses, a small subgroup. For shift-change frequency (panel B), the median score was highest in the subgroup without shift change (0) and lowest in the subgroup with two or more changes (≥2). Panel C displays daily mean scores against daily step counts; the fitted line is included for visualization only and does not account for within-participant clustering.

### 3.3. Factors Associated with Physiological Stress

Results of the multivariable GEE model are presented in Table 2. Age was independently and inversely associated with the daily mean stress score, with each additional year corresponding to a 0.55-point decrease (β = −0.55; 95% CI: −0.86 to −0.23; *p* = 0.001). Shift type was a significant predictor: relative to day-shift work, night-shift work was associated with a markedly lower stress score (β = −11.97; 95% CI: −17.99 to −5.95; *p* < 0.001), whereas evening-shift work did not differ significantly from day shift work (β = 3.58; 95% CI: −3.77 to 10.93; *p* = 0.340). Shift-change frequency showed a comparable pattern: relative to a consistent schedule, two or more changes per month were associated with a substantially lower stress score (β = −14.74; 95% CI: −24.00 to −5.47; *p* = 0.002), while a single change per month showed a non-significant trend in the same direction (β = −7.66; 95% CI: −16.16 to 0.85; *p* = 0.078). Daily step count was positively associated with the stress score, with each additional 1000 steps corresponding to a 1.06-point increase (β = 1.06 per 1000 steps/day; 95% CI: 0.75–1.37; *p* < 0.001). In contrast, administrative position, education level, clinical department, and elapsed study time were not significantly associated with the daily mean stress score.

In sensitivity analyses using alternative specifications (Supplementary Table S3), the positive association between daily step count and the stress score and the lower score on night shifts were consistent across models. In contrast, the associations with age and with shift-change frequency were attenuated when the co-varying scheduling dimension was omitted, and nominally significant department and elapsed-time effects emerged only when shift type was excluded. This pattern indicates that shift type and shift-change frequency share explanatory variance, supporting the fully adjusted model in Table 2.

## 4. Discussion

Using continuous physiological monitoring with a consumer-grade wearable device over 90 days, this study examined the demographic and occupational factors associated with an objective, wearable-derived physiological stress indicator among clinical nurses. The principal findings were that younger age, day-shift work, a non-rotating schedule, and higher daily step count were independently associated with higher scores on the wearable-derived physiological stress indicator. These results are noteworthy because they partly diverge from the widely held assumption that night work and frequent shift rotation impose the greatest occupational burden on nurses. Throughout, these values denote a wearable-derived index of autonomic activation and should not be equated with validated self-report measures of perceived psychological stress.

### 4.1. Shift Type and Physiological Stress

Contrary to the common expectation that night work imposes the greatest occupational burden, night-shift nurses in our cohort exhibited significantly lower daily mean scores on the wearable-derived physiological stress indicator than day-shift nurses. Evening-shift work did not differ significantly from day work. Several mechanisms may contribute to this pattern. First, night shifts in many clinical units involve lower scheduled patient throughput, fewer concurrent administrative and family-related demands, and fewer interruptions from routine daytime activities such as ward rounds, visiting hours, and multidisciplinary meetings. These conditions may be reflected in a calmer autonomic profile during working hours. Second, a healthy-worker and self-selection effect is plausible. Nurses who remain on night shifts may be those who tolerate this pattern relatively well. Third, because the score is derived primarily from HRV and is sensitive to physical activity and circadian phase, the lower activity levels and altered circadian context of night work could influence the derived score independently of subjective strain. A lower score on the wearable-derived physiological stress indicator therefore does not necessarily indicate lower perceived stress or better long-term health. On the contrary, night and rotating shift work have been associated with adverse cardiometabolic and sleep outcomes in previous research [24,25]. Further research is needed to confirm our findings.

### 4.2. Shift-Change Frequency and Physiological Stress

A parallel and equally counterintuitive finding was that nurses with more frequent shift rotation had lower scores on the wearable-derived physiological stress indicator than those on a consistent schedule. The largest reduction was observed among nurses undergoing two or more changes per month. This contrasts with a substantial amount of the literature linking rotating shift work to circadian disruption, poorer sleep, and elevated physiological strain [24,26]. Within our cohort, several factors may help reconcile this discrepancy. Frequently rotating nurses may benefit from greater schedule variety and a more even distribution of high- and low-intensity duties, avoiding sustained exposure to a single demanding pattern. Such nurses may also be assigned to rotation precisely because they are regarded as adaptable. Moreover, because the wearable-derived indicator reflects short-term autonomic balance rather than cumulative circadian disruption, it may not fully capture the longer-term physiological costs that rotating schedules are known to incur. These observations should be regarded as hypothesis-generating rather than as evidence that frequent rotation is protective.

### 4.3. Age, Education, and Role

Age was a robust independent predictor of the wearable-derived physiological stress indicator, with older nurses exhibiting consistently lower scores. This is consistent with the view that accumulated clinical experience fosters more effective coping strategies, greater familiarity with workflow, and enhanced emotional regulation. All of the above may attenuate the autonomic stress response during routine clinical work. By contrast, neither education level nor administrative position was significantly associated with the daily mean stress score. Although deputy head nurses and head nurses did not differ significantly from staff nurses in wearable-derived physiological stress scores, despite the additional managerial and coordination responsibilities of these roles, the small number of nurses in administrative positions (n = 6) limited the precision of this estimate. The absence of an association with education most likely reflects the homogeneous educational profile of the cohort, in which more than nine out of ten nurses held a bachelor’s degree. Similarly, the lack of a department effect (surgical versus emergency) suggests that, within this single institution, the two high-acuity settings imposed broadly comparable physiological demands. Because staffing levels and patient acuity were not directly measured, they may warrant attention in future multi-unit comparisons [27].

### 4.4. Daily Step Count and Physiological Stress

Daily step count was positively associated with the wearable-derived physiological stress indicator, with each additional 1000 steps corresponding to a 1.06-point increase. In a clinical nursing context, step count likely reflects work intensity rather than leisure-time exercise: hospital nurses typically accumulate 8000–13,000 steps per shift through frequent movement between patient rooms, nursing stations, and supply areas, with higher counts on busier shifts [28]. This interpretation is consistent with the lower stress scores observed during night shifts, when ward activity and ambulation are typically reduced [28]. The finding also aligns with the broader “physical activity paradox,” whereby occupational physical activity may impose physiological strain rather than confer health benefits, owing to its sustained intensity and limited recovery [29].

Two caveats temper this interpretation. First, step count and the stress score are both generated by the same wearable. Because physical activity influences heart rate variability, the two measures may share method variance. Although the Firstbeat algorithm suppresses stress scoring during detected activity, which partially decouples them, residual overlap cannot be excluded. The association should therefore not be read as a wholly independent predictor.

Second, step count is multidimensional and may index ward layout, staffing patterns, and patient acuity in addition to workload. Its meaning should be interpreted with corresponding caution. Because the present analysis did not distinguish on-shift from off-duty steps, future studies separating the two would help clarify whether the association reflects occupational workload, physiological arousal from exertion, or both.

### 4.5. Strengths and Limitations

This study has several strengths. To our knowledge, it is among the few investigations to apply continuous, objective physiological monitoring to nurse stress in a clinical setting over an extended 90-day period, generating dense repeated measurements that were appropriately analyzed with a GEE model accounting for within-subject correlation. Reliance on a wearable device reduced the recall bias inherent in questionnaire-based designs. Moreover, the integration of demographic and occupational data from institutional records enabled simultaneous adjustment for multiple work-related factors.

Several limitations must nevertheless be acknowledged.

First, the single-center design with a convenience sample of 52 nurses from the surgical and emergency departments limits statistical power for less common subgroups and constrains the generalizability of the findings to other settings and specialties and other countries and health systems.

Second, the algorithm-derived stress score is based largely on HRV. When benchmarked against an ECG-based reference, this score has been shown to track stress-related autonomic activation in healthy young adults but to correspond only marginally with self-reported perceived stress [17]. Because the algorithm does not expose raw inter-beat-interval data, its output cannot be independently recomputed. Moreover, it has not been independently validated against gold-standard psychophysiological measures specifically in clinical nursing populations. We therefore treat this measure as a wearable-derived physiological stress indicator rather than a validated measure of psychological stress. A concurrent validated self-report measure is needed to relate our findings to subjective experience.

Third, the counterintuitive associations with night work and frequent rotation may partly reflect healthy-worker and self-selection effects that an observational design cannot disentangle. Unmeasured confounders, including patient acuity, staffing ratios, patient-to-nurse ratio, overtime, individual sleep behavior, caffeine and tobacco use, and concurrent medications, were not captured. Professional experience was only partly reflected by age, which was retained in the model.

Fourth, wear-time compliance was higher among surgical than emergency nurses (Supplementary Table S4). This department-level difference represents a potential source of residual selection bias that an observational design cannot fully exclude. Future studies should monitor and report compliance across relevant subgroups.

Beyond statistical significance, the magnitudes warrant consideration. The night-shift and high-rotation coefficients correspond to differences of roughly 12–15 points on the 0–100 scale of the wearable-derived physiological stress indicator. Interpreting the clinical significance of these differences is constrained by the absence of an established minimal clinically important difference for the Garmin/Firstbeat stress score. For reference, the manufacturer divides the 0–100 score into four bands (rest 0–25, low 26–50, medium 51–75, high 76–100). The differences observed here correspond to roughly half of one band and could, in some individuals, shift the classification into an adjacent stress band. Whether a change in this magnitude translates into meaningful differences in fatigue, wellbeing, or health outcomes in everyday nursing practice remains uncertain, and this limitation should be considered when interpreting our findings. Moreover, the per-year age and per-1000-step coefficients are individually small and become clinically meaningful only across wide ranges of age or daily activity.

Finally, because the analysis is observational, the associations reported here should not be interpreted as causal. In addition, although the device also recorded sleep duration and stages, these metrics were not incorporated into the present analysis. Given the centrality of sleep to shift-related strain, future work should model device-derived sleep alongside the wearable-derived physiological stress indicator.

### 4.6. Implications for Practice and Future Research

From a practical standpoint, these findings suggest that occupational-health efforts should not focus exclusively on night and rotating shifts. We should also attend to the physiological burden carried by younger, less experienced nurses and by those on fixed day schedules. For this group, structured mentorship, graduated workload allocation, and targeted coping-skills training may be especially valuable. The demonstrated feasibility of continuous wearable monitoring further supports its use as a low-burden screening and feedback tool.

Future research should build on these findings with larger, multicenter, and longitudinal designs that pair objective wearable measures with validated self-report instruments (e.g., the Perceived Stress Scale, the Nursing Stress Scale, the Depression, Anxiety and Stress Scales, or the Maslach Burnout Inventory).

## 5. Conclusions

Consumer-grade wearable monitoring is a feasible, objective complement to self-report assessment. Younger age, day-shift work, a consistent schedule, and higher daily step count were independently associated with higher scores on the wearable-derived physiological stress indicator in clinical nurses. These associations reflect autonomic physiological activation captured by the device and should not be equated with perceived psychological or work-related stress. The counterintuitive associations observed for night and rotating shifts should be interpreted as hypothesis-generating rather than as evidence that these schedules are protective; they may reflect residual confounding, autonomic adaptation, or a healthy-worker effect and warrant confirmation in larger, longitudinal studies. Our findings require confirmation against validated self-report measures before informing occupational-health decisions. Identifying which nurses and work patterns show the highest indicator scores offers a starting point for more precisely targeted occupational-health strategies.

## Figures and Tables

**Table 1:** Demographic and occupational characteristics of the participating registered nurses (n = 52).

Characteristics	n (%)
Age, median (IQR)	28.5 (25.0–33.0)
**Age group**	
21–25 years	16 (30.8%)
26–30 years	16 (30.8%)
31–35 years	10 (19.2%)
36–40 years	5 (9.6%)
41–55 years	5 (9.6%)
**Gender**	
Male	4 (7.7%)
Female	48 (92.3%)
**Nursing Education**	
Bachelor’s Degree	48 (92.3%)
Master’s/Doctorate Degree	4 (7.7%)
**Department**	
Surgical Department	27 (51.9%)
Emergency Department	25 (48.1%)
**Shift**	
Day Shift	28 (53.8%)
Evening Shift	17 (32.7%)
Night Shift	7 (13.5%)
**Children**	
No	43 (82.7%)
Yes	9 (17.3%)
**Marital Status**	
Single	39 (75.0%)
Married	13 (25.0%)
**Job Title**	
Nurse	46 (88.5%)
Deputy Head Nurse/Head Nurse	6 (11.5%)
Salary, median (IQR)	US$1555 (US$1368–US$1839)
**Salary Range** (**US Dollars**)	
≤$1270	7 (13.5%)
$1271–US$1587	23 (44.2%)
$1588–US$1905	12 (23.1%)
$1906–US$2222	4 (7.7%)
≥$2223	6 (11.5%)
**Steps/day, median** (**IQR**)	7864.0 (5367.0–11,564.3)
**wear-time, day**	59.0 (50.3–85.5)

**Table 2:** Multivariable generalized estimating equation analysis of factors associated with the daily mean physiological stress score.

Variable	β	SE	95% CI	*p*-Value
Age	−0.55	0.16	(−0.86, −0.23)	0.001 **
Shift frequency				
0	Reference			
1	−7.66	4.34	(−16.16, 0.85)	0.078
2+	−14.74	4.73	(−24.00, −5.47)	0.002 **
Shift				
Day	Reference			
Evening	3.58	3.75	(−3.77, 10.93)	0.340
Night	−11.97	3.07	(−17.99, −5.95)	<0.001 **
Position				
Nurse	Reference			
Deputy Head Nurse/Head Nurse	7.07	4.34	(−1.44, 15.59)	0.103
Education				
Bachelor’s degree	Reference			
Master’s/Doctorate	1.12	4.06	(−6.83, 9.08)	0.782
Department				
Surgical	Reference			
Emergency	1.72	3.33	(−4.81, 8.25)	0.606
Time	0.03	0.04	(−0.05, 0.12)	0.424
Steps	1.06	0.16	(0.75, 1.37)	<0.001 **

Outcome: daily mean stress score (0–100) per nurse. A multivariable GEE model with the individual nurse as the subject variable, an exchangeable working correlation structure, and robust standard errors were used to account for repeated daily measurements within individuals. Steps were modeled per 1000 steps/day and time per day of follow-up. β, regression coefficient; SE, standard error; CI, confidence interval; ref., reference category. ** *p* < 0.01.

## Data Availability

The datasets generated and analyzed during the current study are available from the corresponding author upon reasonable request. The data are not publicly available due to privacy and ethical restrictions.

## Supplementary Materials

The following supporting information can be downloaded at https://www.mdpi.com/article/10.3390/healthcare14152282/s1, Table S1. Model fit for candidate generalized estimating equation (GEE) working correlation structures; Table S2. Multicollinearity diagnostics for the predictors entered into the multivariable GEE model; Table S3. Sensitivity analyses using alternative GEE specifications in which shift type and shift-change frequency were entered separately rather than jointly, Model A. Shift type retained; shift-change frequency omitted, Model B. Shift-change frequency retained; shift type omitted.

## Abbreviations

HRV	heart rate variability
HR	heart rate
PPG	photoplethysmography
IQR	interquartile range
GEE	generalized estimating equation
CI	confidence interval
SE	standard error
