# Supplementary material for: Factors Associated with Wearable-Derived Physiological Stress Indicators Among Clinical Nurses: A Longitudinal Observational Study Using Continuous Monitoring
Source: Healthcare (Basel). 2026 Jul 27;14(15):2282. doi: 10.3390/healthcare14152282 (PMC13465576; doi:10.3390/healthcare14152282)
Supplement: Supplementary file 1 [file healthcare-14-02282-s001.zip › healthcare-4383681-supplementary.pdf]

Supplementary Materials

Table S1. Model fit for candidate generalized estimating equation (GEE) working correlation structures.

Working correlation structure	QIC	QICC
Independent	726,198.3	726,096.6
Exchangeable	715,376.6	715,282.6
AR(1)	716,235.6	716,149.9

QIC, quasi-likelihood under the independence model criterion; QICC, corrected QIC. Smaller values indicate better fit. The exchangeable structure (bold) yielded the lowest QIC and QICC and was selected as the primary specification.

Table S2. Multicollinearity diagnostics for the predictors entered into the multivariable GEE model.

Variable	Tolerance	VIF
Age	0.477	2.096
**Shift**		
Day shift	Reference	
Evening shift	0.791	1.264
Night shift	0.770	1.298
**Shift frequency**		
0	Reference	
1	0.490	2.042
2+	0.564	1.772
**Position**		
Nurse	Reference	
Deputy Head Nurse / Head Nurse	0.152	6.589
**Education**		
Bachelor's degree	Reference	
Master's / Doctorate	0.188	5.333
**Department**		
Surgical department	Reference	
Emergency department	0.783	1.277
Steps	0.976	1.024

VIF, variance inflation factor. Collinearity statistics were obtained from an auxiliary ordinary least-squares regression on the same set of predictors. All predictors showed tolerance > 0.1 and VIF < 10; the overall condition index was below 30, indicating no problematic multicollinearity.

Table S3. Sensitivity analyses using alternative GEE specifications in which shift type and shift-change frequency were entered separately rather than jointly

Model A. Shift type retained; shift-change frequency omitted

**Variable**	**β**	**SE**	**95% CI**	***p***
Age	−0.30	0.32	(−0.93, 0.33)	0.356
Shift				
Day shift	Reference			
Evening shift	7.26	4.36	(−1.28, 15.80)	0.096
Night shift	−9.24	2.80	(−14.73, −3.75)	<0.001**
Position				
Nurse	Reference			
Deputy/head nurse	−1.73	3.54	(−8.67, 5.21)	0.625
Department				
Surgical department	Reference			
Emergency department	3.42	3.43	(−3.29, 10.14)	0.318
Time	0.06	0.04	(−0.01, 0.13)	0.093
Steps (per 1,000/day)	1.20	0.15	(0.90, 1.50)	<0.001**

Model B. Shift-change frequency retained; shift type omitted

**Variable**	**β**	**SE**	**95% CI**	***p***
Age	−0.60	0.36	(−1.30, 0.11)	0.099
Shift frequency				
0	Reference			
1	1.58	5.51	(−9.22, 12.39)	0.774
2+	−6.15	5.13	(−16.22, 3.91)	0.231
Position				
Nurse	Reference			
Deputy/head nurse	−8.35	4.62	(−17.40, 0.70)	0.070
Department				
Surgical department	Reference			
Emergency department	9.20	4.03	(1.31, 17.09)	0.022*
Time	0.10	0.03	(0.04, 0.16)	0.001**
Steps (per 1,000/day)	0.93	0.17	(0.59, 1.27)	<0.001**

Outcome: daily mean stress score, 0–100

All models used a GEE with an exchangeable working correlation structure and robust (sandwich) standard errors, with the individual nurse as the subject variable. Steps were modeled per 1,000 steps/day and time per day of follow-up. β, regression coefficient; SE, standard error; CI, confidence interval. **p* < 0.05; ***p* < 0.01.

Table S4. Association between wear-time compliance and participant and work characteristics.

Characteristic	Median (IQR) / ρ	p value
**Shift**		**0.773**
Day	0.66 (0.57–0.95)	
Evening	0.66 (0.53–0.88)	
Night	0.80 (0.41–0.97)	
**Department**		**<0.001****
Surgical	0.93 (0.66–0.97)	
Emergency	0.60 (0.47–0.66)	
Age †	−0.084	0.552
Workload †	0.133	0.347

Shift and Department were compared using the Kruskal–Wallis test; values are the median (IQR) of wear-time compliance (proportion of the monitoring period with valid data).

†Age and Workload were assessed using the Spearman rank correlation coefficient (ρ). ** $p < 0.01$.
